# Preliminary Quality Evaluation and Characterization of Phenolic Constituents in Cynanchi Wilfordii Radix

**DOI:** 10.3390/molecules23030656

**Published:** 2018-03-14

**Authors:** Takashi Uchikura, Hiroaki Tanaka, Hidemi Sugiwaki, Morio Yoshimura, Naoko Sato-Masumoto, Takashi Tsujimoto, Nahoko Uchiyama, Takashi Hakamatsuka, Yoshiaki Amakura

**Affiliations:** 1 Department of Pharmacognosy, College of Pharmaceutical Sciences, Matsuyama University, 4-2 Bunkyo-cho, Matsuyama, Ehime 790-8578, Japan; 46150019@g.matsuyama-u.ac.jp (T.U.); 16130815@g.matsuyama-u.ac.jp (H.T.); h_sugiwa@g.matsuyama-u.ac.jp (H.S.); myoshimu@g.matsuyama-u.ac.jp (M.Y.); 2 Division of Pharmacognosy, Phytochemistry and Narcotics, National Institute of Health Sciences, 3-25-26 Tonomachi, Kawasaki-ku, Kawasaki, Kanagawa 210-9501, Japan; nasato@nihs.go.jp (N.S.-M.); tsujimoto@nihs.go.jp (T.T.); nuchiyama@nihs.go.jp (N.U.); thakama@nihs.go.jp (T.H.)

**Keywords:** *Cynanchum wilfordii*, phenolic glycoside, 2-*O*-β-laminaribiosyl-4-hydroxyacetophenone, cynandione A, thin layer chromatography, *Cynanchum auriculatum*

## Abstract

A new phenolic compound, 2-*O*-β-laminaribiosyl-4-hydroxyacetophenone (**1**), was isolated from Cynanchi Wilfordii Radix (CWR, the root of *Cynanchum wilfordii* Hemsley), along with 10 known aromatic compounds, including cynandione A (**2**), bungeisides-C (**7**) and –D (**8**), *p*-hydroxyacetophenone (**9**), 2′,5′-dihydroxyacetophenone (**10**), and 2′,4′-dihydroxyacetophenone (**11**). The structure of the new compound (**1**) was elucidated using spectroscopic methods and chemical methods. The structure of cynandione A (**2**), including a linkage mode of the biphenyl parts that remained uncertain, was unambiguously confirmed using the 2D ^13^C–^13^C incredible natural abundance double quantum transfer experiment (INADEQUATE) spectrum. Additionally, health issues related to the use of Cynanchi Auriculati Radix (CAR, the root of *Cynanchum auriculatum* Royle ex Wight) instead of CWR have emerged. Therefore, constituents present in methanolic extracts of commercially available CWRs and CARs were examined using UV-sensitive high-performance liquid chromatography (HPLC), resulting in common detection of three major peaks ascribed to cynandione A (**2**), *p*-hydroxyacetophenone (**9**), and 2′,4′-dihydroxyacetophenone (**11**). Thus, to distinguish between these ingredients, a thin-layer chromatography (TLC) method, combined with only UV irradiation detection, focusing on wilfosides C1N (**12**) and K1N (**13**) as marker compounds characteristic of CAR, was performed. Furthermore, we propose this method as a simple and convenient strategy for the preliminary distinction of CWR and CAR to ensure the quality and safety of their crude drugs.

## 1. Introduction

Cynanchi Wilfordii Radix (CWR), the dried root of *Cynanchum wilfordii* Hemsley (family Asclepiadaceae), is a crude drug listed in the Korean Herbal Pharmacopoeia [1]. CWR has been used in Korea as a substitute for Polygoni Multiflori Radix, the dried root of *Polygonum multiflorum* Thunberg (Polygonaceae), which is used for its restorative effects and is one of the important crude drugs listed in the Japanese, Korean, and Chinese Pharmacopoeias. Recently, the use of Cynanchi Auriculati Radix (CAR), the dried root of *Cynanchum auriculatum* Royke ex Wight, instead of CWR has led to health problems in Korea [2,3]. Although CAR resembles CWR closely in appearance, CAR, a crude drug that differs from CWR in China, is currently treated as a toxic plant by the U.S. Food and Drug Administration (FDA) [4]. Therefore, standards and methods to distinguish CWR from CAR should be established to ensure the quality and safety of the crude drugs. We recently reported a survey on the original plant species of crude drugs widely distributed as CWR in the Korean and Chinese markets. This study revealed that CAR was incorrectly used in eight of the 13 products distributed as CWR, including possible confusion of CWR and CAR [5]. Previous phytochemical investigations of CWR identified the presence of pregnane glycosides, acetophenones, and humulanolides [6,7,8,9,10,11]. Although there are several reports on ingredient research using materials of CWA and CAR available on the market, they may not be of the precise species. Therefore, detailed phytochemical information on the raw material with defined origins is necessary to ensure the quality and safety of crude drugs.

Several studies have assessed the quality of CWR and CAR and have aimed to distinguish between them using high-performance liquid chromatography (HPLC) [12,13], and many methods used to identify crude drugs ensure reliability. However, in this study, the characterization of phenolic constituents in authentic original CWR plant species is identified using DNA sequences [5], and a simple and convenient thin-layer chromatography (TLC) method for the distinction of CWR and CAR to ensure the quality and safety of their crude drugs.

## 2. Results and Discussion

### 2.1. Isolation and Characterization

**Figure 1:**
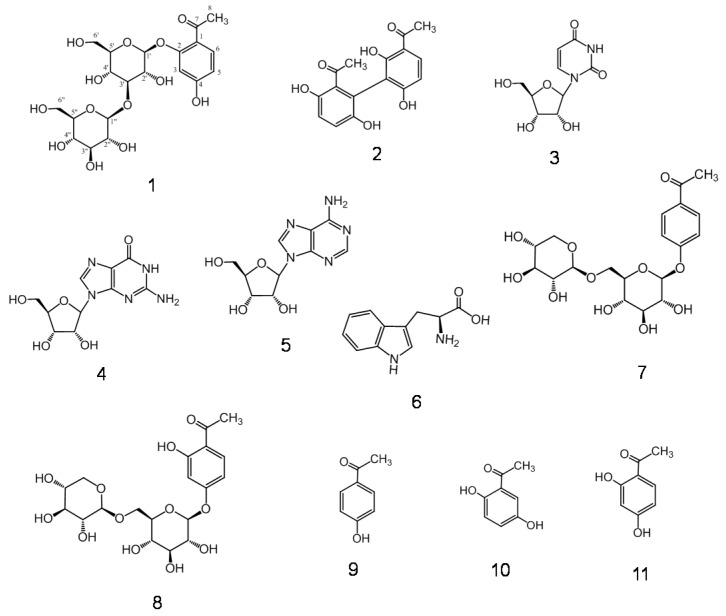
Structures of compounds **1**–**11**.

A homogenate of CWR in 80% methanol (MeOH) was concentrated and further extracted with *n*-hexane, ethyl acetate (EtOAc), and *n*-butanol (BuOH) to obtain the respective extracts and water (H_2_O) extract. HPLC analysis was used to monitor the ultraviolet (UV)-sensitive compounds (phenols) in the EtOAc and *n*-BuOH extracts, which were separately chromatographed using a Diaion HP-20, YMC GEL ODS-AQ, and Chromatorex ODS with MeOH-H_2_O in a stepwise gradient mode. The fractions showing similar HPLC or TLC patterns were combined and further purified using column chromatography to obtain compound **1**, cynandionene A (**2**) [14], uridine (**3**), guanosine (**4**), adenosine (**5**), tryptophan (**6**) [15], bungeiside-C (**7**), bungeiside-D (**8**), *p*-hydroxyacetophenone (**9**) [16], 2′,5′-dihydroxyacetophenone (**10**) [13], and 2′,4′-dihydroxyacetophenone (**11**) [16]. The known compounds **2**–**11** were identified by direct comparison with authentic specimens and by comparing their spectral data with those reported in the literature (Figure 1).

Compound **1** was isolated as a light brown amorphous powder. Its molecular formula was assigned as C_2__0_H_28_O_1__3_ based on its high resolution-electrospray ionization (HR-ESI)-mass spectrometry (MS, *m*/*z* 475.1473 [M–H]^−^; calcd. for C_2__0_H_28_O_1__3_-H: 475.1457) and ^13^C-NMR (20 ^13^C signals) spectra. The UV spectrum showed absorption maxima at 228, 269, and 301 nm. The proton (^1^H)- and ^13^C-NMR spectra of compound **1** exhibited the following signal characteristics of the 2′,4′-hydroxyacetophenone moiety. The ^1^H-NMR spectrum (Figure S1) assigned based on the ^1^H-^1^H correlation spectroscopy (COSY) (Figure S2) exhibited signals due to an acetyl group (δ_H_ 2.62, 3H) and ABX-type proton signals due to a trisubstituted benzene proton, δ_H_ 6.69 (d, *J* = 2.0 Hz), 6.50 (dd, *J* = 2.0, 8.5 Hz), and 7.68 (d, *J* = 8.5 Hz), and two sets of sugar protons.

**Figure 2:**
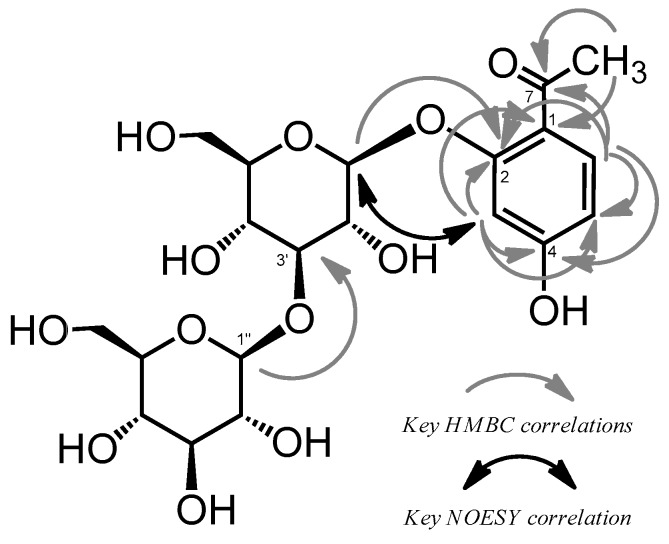
Key heteronuclear multiple bond connectivity (HMBC) correlations and nuclear Overhause effect spectroscopy (NOESY) correlation of compound **1**.

This acetophenone unit was also supported by eight carbon signals, δ_C_ 121.3, 160.9, 103.7, 164.9, 110.8, 133.3, 200.3, and 32.1 (C-1–8), in the ^13^C-NMR spectrum (Figure S3) assigned based on heteronuclear single quantum coherence (HSQC) and heteronuclear multiple bond connectivity (HMBC) spectra (Figure 2, Figures S4 and S5). Additionally, an aglycone of compound **1** was chemically substantiated by acid hydrolysis followed by HPLC analysis, which showed the production of 2′,4′-dihydroxyacetophenone. The presence of two sugar units in **1** was indicated by two anomeric proton signals at δ_H_ 5.05 (d, *J* = 7.5 Hz) and 4.58 (d, *J* = 7.5 Hz) and others assigned based on COSY, as shown in Table 1. Thus, the sugar residues were presumed to be hexoses, as revealed by 12 aliphatic carbon signals (δ_C_ 102.1, 110.8, 74.1, 75.5, 88.2, 77.9, 69.7, 71.6, 78.0, 78.2, 62.4, and 62.6) in the ^13^C-NMR spectrum.

The sugar unit obtained following acid hydrolysis of compound **1** was identified as d-glucose by the HPLC analysis of derivatives prepared by the reaction with l-cysteine methyl ester and *o*-tolyl isothiocyanate according to the previously reported method [17]. The linking position of each unit was determined by correlations among the glucose H-1′ (δ 5.05)/C-2 (δ 160.9) of the acetophenone moiety and glucose H-1″ (δ 4.95)/glucose C-3 (δ 88.2) in the HMBC spectrum. Moreover, the nuclear Overhause effect spectroscopy (NOESY) results showed a correlation between the glucose H-1′ (δ_H_ 5.05) and H-3 (δ_H_ 6.69) (Figure 2). β-Glycosidic linkages at each glucose core were assigned by a large coupling constant (*J* = 7.5 Hz). Therefore, compound **1** was established as 2-*O*-β-laminaribiosyl-4- hydroxyacetophenone.

**Figure 3:**
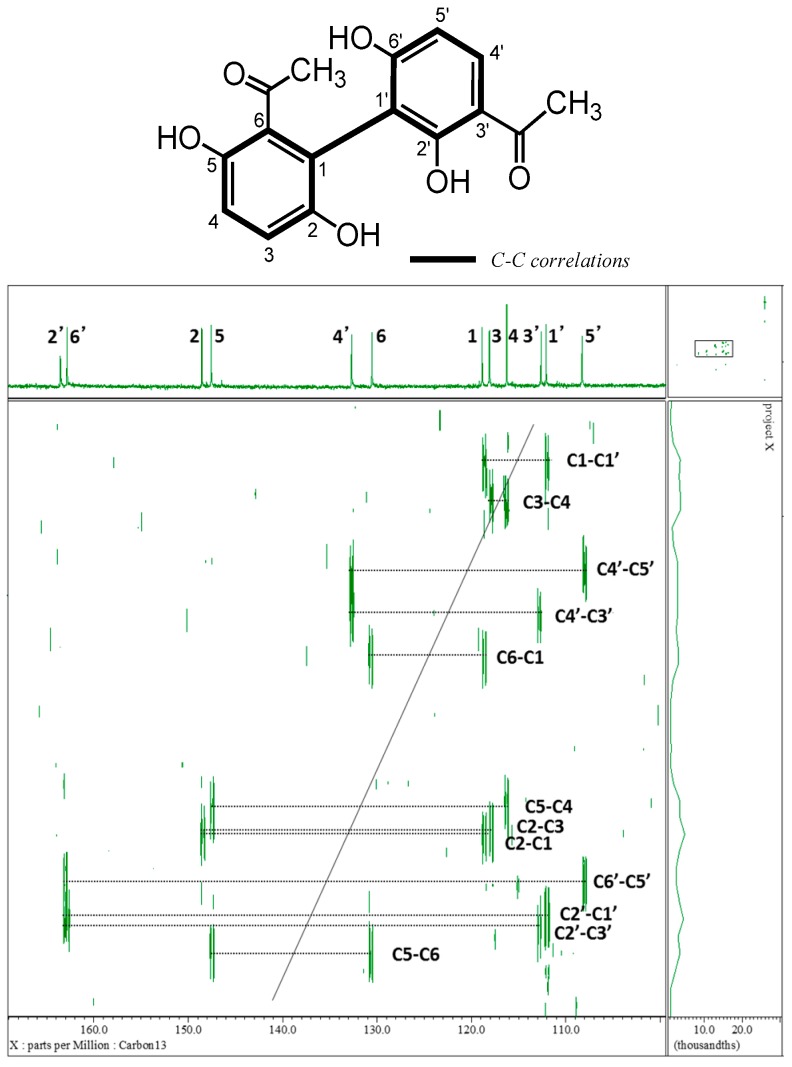
Two-dimensional incredible natural abundance double quantum transfer experiment (2D-INADEQUATE) spectrum of compound **2**.

Cynandionene A (**2**) is a characteristic biacetophenone derivative with a biphenyl structure, which was revised from 4,3′-diacetyl-2,3,2′,6′-tetrahydrohydroxybiphenyl by 6,3′-diacetyl-2,5,2′,6′- tetrahydrohydroxybiphenyl after the structural elucidation [14]. It was difficult to confirm the present structure based only on the HMBC spectrum because a connection between C-1 and C-1′ could not be confirmed. Therefore, in this study, we attempted to prove the connection of the biphenyl carbon–carbon bond using two-dimensional (2D) incredible natural abundance double quantum transfer experiment (INADEQUATE) for the first time. All C–C correlations were observed as shown in Figure 3. Compound **2** was shown to have C–C correlations between C-1 and C-1′. Therefore, the present biphenyl structure of compound **2** was supported by the 2D-INADEQUATE data. The positions of two acetyl groups were also confirmed using HMBC.

### 2.2. Preliminary Quality Evaluation of CWR and CAR Using TLC

**Figure 4:**
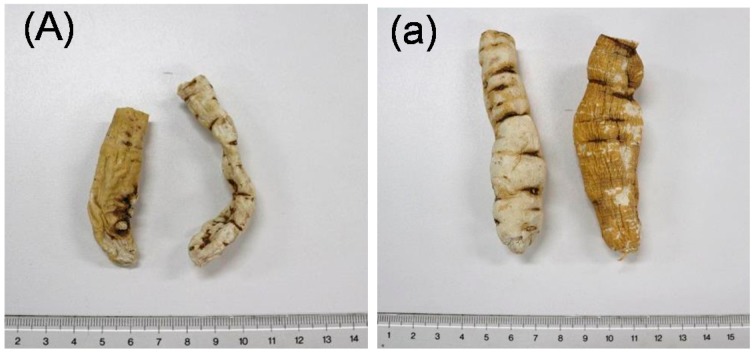
Crude drugs identified: (**A**) Cynanchi Wilfordii Radix (CWR, the root of *Cynanchum wilfordii* Hemsley) (Product C), and (**a**) Cynanchi Auriculati Radix (CAR, the root of *Cynanchum auriculatum* Royke ex Wight) (Product h).

**Figure 5:**
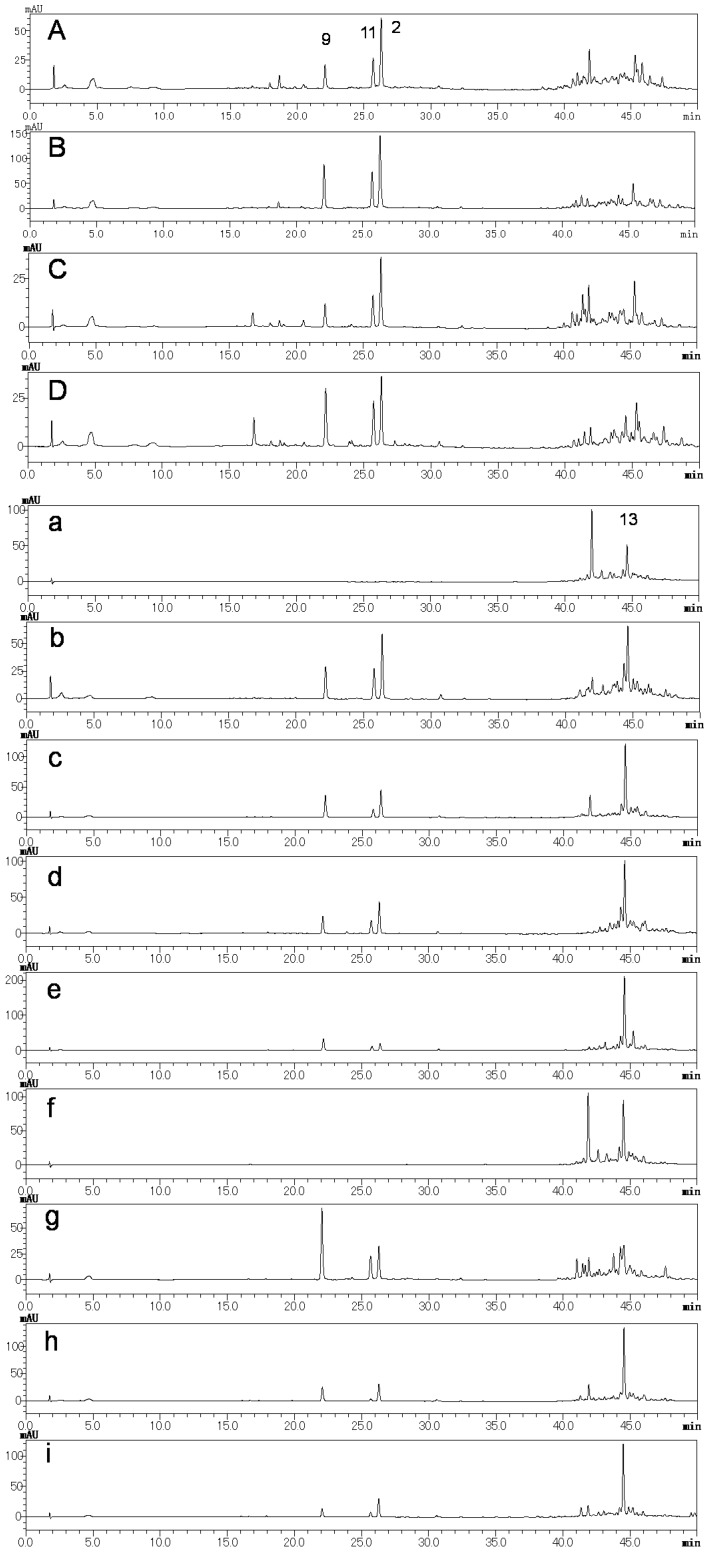
HPLC chromatograms of the crude drug extracts identified as Cynanchi Wilfordii Radix (CWR) (**A**–**D**) and Cynanchi Auriculati Radix (CAR) (**a**–**i**). The number on the chromatogram corresponds to the compound number. HPLC conditions are described in condition 1 of Section 3.

CAR resembles CWR closely in appearance as shown in Figure 4. In this study, CWR and CAR (four and nine samples, respectively) identified using DNA sequences [5] were used as the test samples (Table 2 and Figure 4). HPLC chromatograms of MeOH extracts (CWR-Ex and CAR-Ex) obtained from these samples are shown in Figure 5.

**Figure 6:**
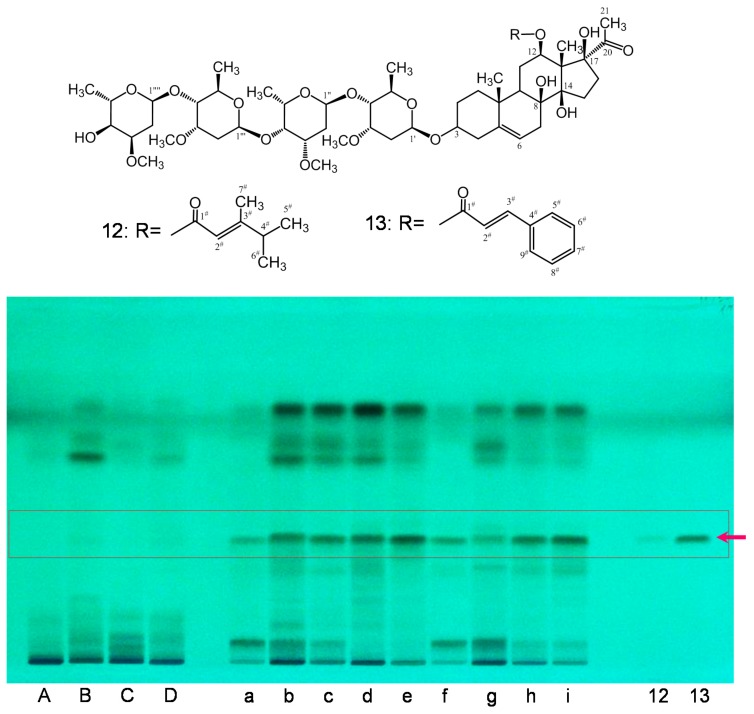
TLC chromatograms of the crude drugs Cynanchi Wilfordii Radix (CWR) (**A**–**D**) and Cynanchi Auriculati Radix (CAR) (**a**–**i**). TLC plate illuminated with UV 254 nm. 12: wilfoside C1N, 13: wilfoside K1N. Conditions are described in Section 3.

In all the HPLC analyses of CWR-Ex samples, three main peaks corresponding to cynandione A (**2**), *p*-hydroxyacetophenone (**9**), and 2′,4′-dihydroxyacetophenone (**11**) were detected. In CAR-Ex, products **b**–**e** and **g**–**i**, but not **a** and **f**, were also mainly detected, suggesting that it would be difficult to distinguish these crude drugs by detecting these three compounds as reference compounds. On the other hand, CAR exhibited a peak corresponding to wilfosides K1N (**13**), which was not clearly detected in those of CWR. Because it was difficult to distinguish the species using HPLC analyses, a TLC method was developed. The TLC chromatogram of CAR-Ex with an EtOAc/water/MeOH/acetic acid (200:10:10:3, *v*/*v*/*v*/*v*) solvent mixture (A) as the mobile phase provided well-separated spots under UV light (254 nm) including a clear spot with approximately *R*f 0.5 (Figure 6). This spot was revealed to be due to two compounds with almost the same *R*fs. These two compounds were isolated by preparative TLC with the other solvent system, *n*-hexane-acetone (1:1), leading to clearly separated spots and were identified as wilfosides C1N (**12**) and K1N (**13**) [18].

Several previous studies have reported strategies for distinguishing CWR and CAR. For example, one method evaluated seven compounds in each sample, whereas another study used conduritol F as a marker compound, which is a characteristic constituent in CWR [12,13]. However, one method was complicated because it required the analysis of numerous constituents in samples using HPLC, and the other involved detection using a spray reagent using TLC. Additionally, the identification of the type of samples is vague, although the appearances are similar. The method proposed in the present study is extremely simple because the sample was extracted with MeOH, followed only by a TLC method with UV irradiation detection. Thus, this method could be useful as a distinguishing tool among the preliminary TLC methods for comparison of CWR and CAR.

## 3. Experimental Section

### 3.1. General

Optical rotations were measured using a JASCO P-1020 digital polarimeter (JASCO Corporation, Tokyo, Japan). The UV spectra were recorded using a Shimadzu UVmini-1240 (Shimadzu Corporation, Kyoto, Japan). The HR-ESI-MS spectra were obtained using a micrOTOF-Q (Bruker Daltonics, Billerica, MA, USA) mass spectrometer with acetonitrile as the solvent. The NMR spectra were recorded using a Bruker AVANCE500 instrument (Bruker BioSpin, Billerica, MA, USA; 500 and 126 MHz for ^1^H and ^13^C, respectively) and chemical shifts were expressed as parts per million (ppm) relative to those of the solvents [MeOH-*d*_4_ (δ_H_ 3.30; δ_C_ 49.0), and dimethyl sulfoxide (DMSO)-*d*_6_ (δ_H_ 2.50; δ_C_ 39.5)] on a tetramethylsilane scale. The standard pulse sequences programmed for the instrument (AVANCE 500) were used for each 2D measurement (COSY, HSQC, and HMBC). The 2D-INADEQUATE spectrum was recorded using a JEOL ECA800 instrument (JEOL, Tokyo, Japan). Column chromatography was carried out using the Diaion HP-20, MCI-gel CHP-20P (Mitsubishi Chemical Co., Tokyo, Japan), Chromatorex ODS (Fuji Silysia Chemical Ltd., Aichi, Japan) and YMC GEL ODS (YMC Co. Ltd., Kyoto, Japan), respectively. Preparative TLC was carried out using TLC Silica gel 60 F_254_ glass plates (Merck, Darmstadt, Germany). TLC was performed with CAMAG HPTLC equipment (CAMAG, Muttenz, Switzerland) including a Linomat V applicator (CAMAG) and visualizer documentation system (CAMAG). The samples were spotted on HPTLC Silica gel 60 F_254_ glass plates (20 × 10 cm, Merck), and the spots were detected using UV irradiation at 254 nm. The reversed-phase (RP) HPLC conditions were as follows. Condition 1: column, l-column ODS (5 μm, 150 × 2.1 mm i.d., Chemicals Evaluation and Research Institute, Tokyo, Japan); mobile phase, solvent A was 0.1% formic acid in water, and solvent B was 0.1% formic acid in acetonitrile (0–30 min, 0–50% B in A; 30–35 min, 50–85% B in A; 35–40 min, 85–85% B in A); injection volume, 2 μL; column temperature, 40 °C; flow-rate, 0.3 mL/min; and detection wavelength, 200–400 nm. Condition 2: column, YMC-pack ODS-AQ-3C2 (5 μm, 150 × 2.0 mm i.d., YMC Co. Ltd., Kyoto, Japan); mobile phase, 10 mmol/L phosphoric acid (H_3_PO_4_)-10 mmol/L monopotassium phosphate (KH_2_PO_4_)-acetonitrile (8:2); column temperature, 30 °C; flow-rate, 0.25 mL/min; and detection wavelength, 280 nm. Condition 3: column, YMC-pack ODS-AQ-3C2 (5 μm, 150 × 2.0 mm i.d., YMC Co. Ltd., Kyoto, Japan); mobile phase, 50 mmol/L phosphate buffer-acetonitrile (75:25); column temperature, 35 °C; flow-rate, 0.3 mL/min; and detection wavelength, 250 nm.

### 3.2. Materials

The CWR products used in the phytochemical investigation were purchased at a crude drug store at the Gyeongdong Market (Seoul, Korea). CWR and CAR for HPLC and TLC analyses were obtained from the Gyeongdong or Chinese markets and were provided by Japanese crude drug wholesalers. The identities of all the crude drugs were confirmed using DNA sequences [5]. All other reagents used were of analytical grade.

### 3.3. Extraction and Isolation

The CWR product (300 g) was homogenized in 3 L 80% MeOH [MeOH-H_2_O (8:2)], the homogenate was filtered, concentrated to approximately 300 mL, and then extracted with 3 L each of *n*-hexane, EtOAc, and *n*-BuOH to obtain extracts at yields of *n*-hexane (492.9 mg), EtOAc (7.0 g), *n*-BuOH (13.2 g), and water (43.2 g), respectively. The EtOAc extract (500 mg) was chromatographed using the YMC GEL ODS with MeOH-H_2_O (10:90→20:80→30:70→40:60→50:50→100:0) in stepwise gradient mode. The fractions showing similar HPLC patterns were combined and further purified using column chromatography with the Chromatorex ODS or preparative TLC with *n*-hexane-acetone (2:1 or 1:1), or both to obtain *p*-hydroxyacetophenone (**9**, 13.8 mg), 2′,5′- dihydroxyacetophenone (**10**, 1.0 mg), 2′,4′-dihydroxyacetophenone (**12**, 3.8 mg), and cynandionene A (**2**, 9.6 mg). The *n*-BuOH extract (12.5 g) was similarly separated using column chromatography over Diaion HP-20 with MeOH-H_2_O (0:100→10:90→20:80→30:70→40:60→50:50→ 100:0) in stepwise gradient mode. The H_2_O eluate (3.0 g) was separated using column chromatography using the Chromatorex ODS with aqueous MeOH to obtain uridine (**3**, 6.3 mg), and adenosine (**5**, 2.3 mg). The 10, 20, 30, and 40% MeOH eluates (120, 130, 89, and 52 mg, respectively) were purified using column chromatography with YMC GEL ODS using aqueous MeOH to obtain guanosine (**4**, 21.8 mg), tryptophan (**6**, 20.6 mg), bungeiside-C (**7**, 13.7 mg) plus compound **1** (2.4 mg), and bungeiside-D (**8**, 7.0 mg), respectively. These compounds were identified by direct comparison with authentic specimens or by comparing their spectral data with those reported in the literature. The physical spectral data of the new compound **1** are as follows.

*2-O-β-Laminaribiosyl-4-hydroxyacetophenone* (**1**): A light brown amorphous powder. UV λ_max_ (MeOH) nm (log ε): 228 (3.01), 269 (3.14), 301 (2.94). [α]_D_^24^ -14° (*c* 1.0, MeOH). ^1^H-NMR (500 MHz, MeOH-*d*_4_) and ^13^C-NMR (126 MHz, MeOH-*d*_4_) data are shown in Table 1. HR-ESI-MS *m*/*z*: 475.1473 ([M–H]^−^, Calcd. for C_2__0_H_28_O_1__3_-H: 475.1457).

*Cynandione A* (**2**): ^1^H-NMR (DMSO-*d*_6_, 800 MHz) δ 12.86 (1H, s, 2′-OH), 10.31 (1H, s, 6′-OH), 9.31 (1H, s, 2-OH), 8.49 (1H, s, 5-OH), 7.68 (1H, d, *J* = 2 Hz, H-4′), 6.72 (1H, d, *J* = 8 Hz, H-3), 6.67 (1H, d, *J* = 8 Hz, H-4), 6.43 (1H, d, *J* = 8 Hz, H-5′), 2.50 (3H, s, 8-CH_3_), 2.19 (3H, s, 8′-CH_3_). ^13^C-NMR (DMSO-*d*_6_, 200 MHz) δ 203.7 (C-7′), 203.6 (C-7), 163.0 (C-2′), 162.8 (C-6′), 148.5 (C-2), 147.5 (C-5), 132.7 (C-4′), 130.7 (C-6), 118.7 (C-1), 117.9 (C-3), 116.3 (C-4), 112.8 (C-3′), 112.0 (C-1′), 108.0 (C-5′), 31.2 (C-8), 26.7 (C-8′).

The CAR product (103 g) was homogenized in 80% MeOH (1 L), and the homogenate was filtered, concentrated to approximately 100 mL, and then extracted with *n*-hexane (300 mL), EtOAc (300 mL), *n*-BuOH (3 L), and water to obtain the solvent extracts at yields of 492.9 mg, 7.0 g, 13.2 g, and 43.2 g, respectively. The EtOAc extract (100 mg) was dissolved in MeOH and subjected to preparative TLC [*n*-hexane-acetone (1:1)] to yield wilfoside C1N (**12**, 8.0 mg) and wilfoside K1N (**13**, 10.5 mg). These compounds were identified by comparing their ^1^H- and ^13^C-NMR data with those reported in literatures and were used as standard samples.

### 3.4. Partial Acid Hydrolysis of Compound ***1***

A solution of compound **1** (0.2 mg) in H_2_O (0.2 mL) and 1 mol/L hydrochloric acid (HCl, 0.1 mL) was heated in a boiling water bath for 8 h. After removing the solvent, the residue was analyzed using HPLC (under Condition 2), and 2′,4′-dihyroxyaetophenone was detected.

### 3.5. Determination of Sugar Configuration of Compound ***1***

The sugar configuration was determined using a previously described method [17]. Compound **1** (1.0 mg) was hydrolyzed by heating in 1 mol/L HCl (0.2 mL) and neutralized with Amberlite IRA400. After evaporation, the residue was dissolved in pyridine (0.2 mL) containing l-cysteine methyl ester hydrochloride (1.0 mg) and heated at 60 °C for 1 h. *o*-Tolyl isothiocyanate (1.0 mg) in pyridine (0.2 mL) was then added to the mixture and heated at 60 °C for 1 h. The reaction mixture was directly analyzed using RP-HPLC (under Condition 3). The peak coincided with that of the derivative of the authentic d-glucose sample.

### 3.6. Preparation of Test Solution of the Crude Drugs for HPLC and TLC

A sample of each product obtained from the open market was pulverized (0.2 and 1 g for the HPLC and TLC analyses, respectively), extracted with MeOH (1.0 mL) by sonication for 5 min, centrifuged, and the supernatant obtained was used as the test solution. The HPLC was performed under Condition 1 described in Section 3.1. For the TLC, aliquots (5 µL) of each test solution were applied to the HPTLC plates, which were developed in a TLC chamber saturated with EtOAc/water/MeOH/acetic acid (200:10:10:3, *v*/*v*/*v*/*v*) mixture as the mobile phase. The spots were detected under a UV lamp at 254 nm.

## 4. Conclusions

In the present study, a new phenolic compound, 2-*O*-β-laminaribiosyl-4-hydroxyacetophenone (**1**), was successfully isolated from CWR, in addition to 11 known compounds. Cynandione A (**2**), which is one of the main constituents of CWR with a biphenyl moiety, was identified using its 2D ^13^C–^13^C INADEQUATE spectrum; the carbon–carbon connection of the biphenyl moiety was clearly confirmed for the first time. The component distributions of MeOH extracts of CWR using a UV-sensitive HPLC analysis revealed three peaks of cynandione A (**2**), *p*-hydroxyacetophenone (**9**), and 2′,4′-dihydroxyacetophenone (**11**), which were the main detected constituents. The emerging use of CAR in place of CWR has led to the need for a differentiating method, and therefore, we proposed and developed the present TLC method for the preliminary distinction between CWR and CAR.

## Figures and Tables

**Table 1:** ^1^H- (500 MHz) and ^13^C-NMR (126 MHz) data of compound **1** measured in MeOH-*d*_4_.

Positions	δ_C_	δ_H_ (*J* in Hz)
1	121.3	
2	160.9	
3	103.7	6.69 (d, *J* = 2.0)
4	164.9	
5	110.8	6.50 (dd, *J* = 2.0, 8.5)
6	133.3	7.68 (d, *J* = 8.5)
7	200.3	
8	32.1	2.62 (3H, s)
Glucose-1′	102.1	5.05 (d, *J* = 7.5)
2′	74.1	3.74 (m) ^d^
3′	88.2	3.67 (t, *J* = 9.0)
4′	69.7	3.54 (t, *J* = 9.0)
5′	78.0 ^a^	3.52 (m) ^d^
6′	62.4 ^b^	3.94 (dd, *J* = 1.5, 12.0) ^c^, 3.75 (m) ^c,d^
Glucose-1″	105.3	4.58 (d, *J* = 7.5)
2″	75.5	3.30 (m) ^d^
3″	77.9 ^a^	3.39 (t, *J* = 9.5)
4″	71.6	3.30 (m) ^d^
5″	78.2 ^a^	3.35 (m) ^d^
6″	62.6 ^b^	3.89 (dd, *J* = 2.0, 11.5) ^c^, 3.63 (m) ^c,d^

^a,b,c^ Assignments may be interchanged. ^d^ Overlapped signals.

**Table 2:** The Korean and Chinese market samples used in this study.

Products	Crude Drug	Locality	Market
**A**	Cynanchi Wilfordii Radix (CWR)	Korea	Korea
**B**	Cynanchi Wilfordii Radix (CWR)	Korea	Korea
**C**	Cynanchi Wilfordii Radix (CWR)	Yeongcheon	Korea
**D**	Cynanchi Wilfordii Radix (CWR)	Yeongcheon	Korea
**a**	Cynanchi Auriculati Radix (CAR)	Jiangsu	China
**b**	Cynanchi Auriculati Radix (CAR)	Jiangsu	China
**c**	Cynanchi Auriculati Radix (CAR)	Jiangsu	China
**d**	Cynanchi Auriculati Radix (CAR)	Jiangsu	China
**e**	Cynanchi Auriculati Radix (CAR)	Jiangsu	China
**f**	Cynanchi Auriculati Radix (CAR)	Jiangsu	China
**g**	Cynanchi Auriculati Radix (CAR)	Jiangsu	China
**h**	Cynanchi Auriculati Radix (CAR)	Korea	Korea
**i**	Cynanchi Auriculati Radix (CAR)	Korea	Korea

## Supplementary Materials

The following are available online at www.mdpi.com/xxx/link, Figures S1–S5.
