# Supplementary material for: Preliminary Quality Evaluation and Characterization of Phenolic Constituents in Cynanchi Wilfordii Radix
Source: Molecules. 2018 Mar 14;23(3):656. doi: 10.3390/molecules23030656 (PMC6017071; doi:10.3390/molecules23030656)
Supplement: Supplementary file 1 [file molecules-23-00656-s001.pdf]

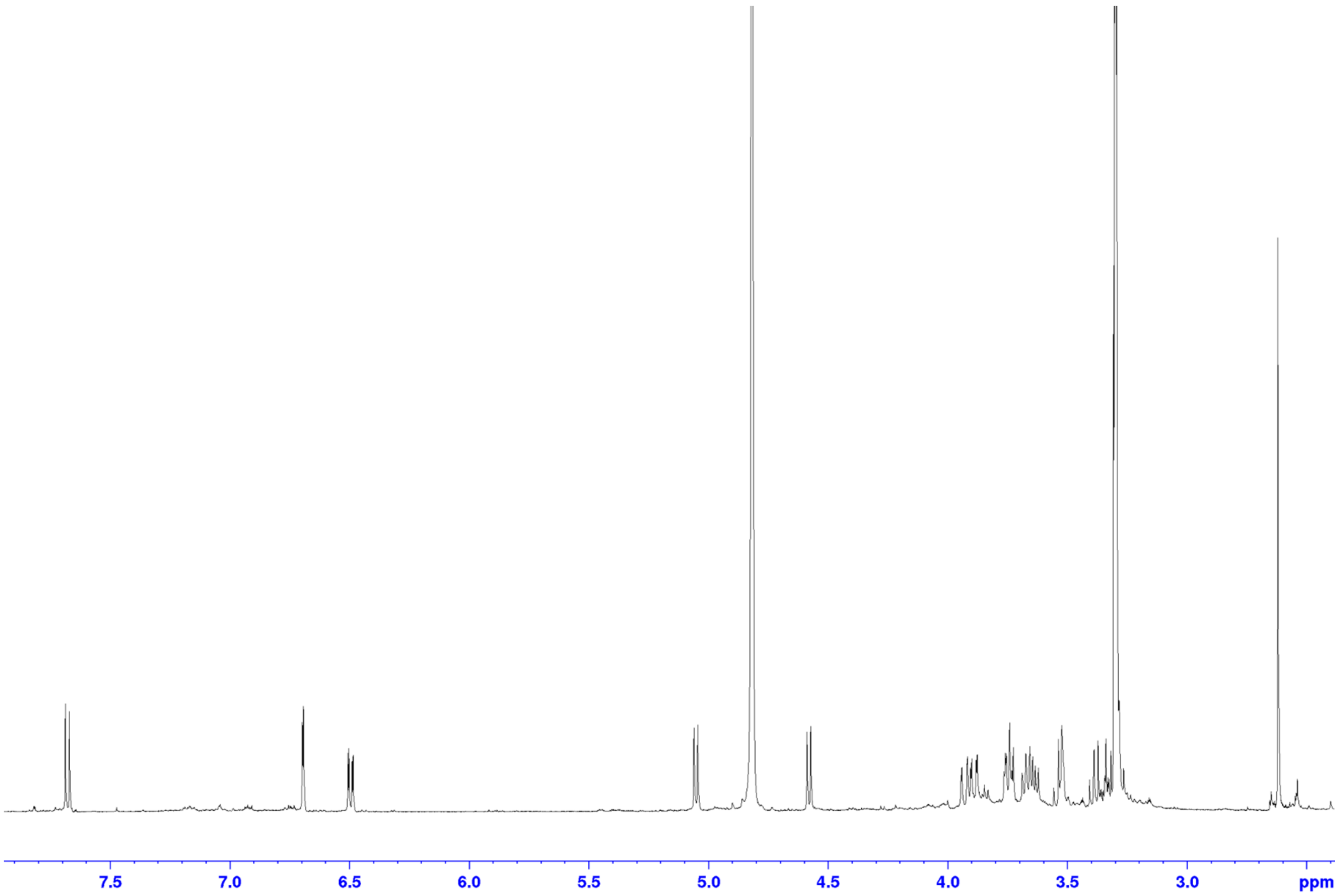

Figure S1. ^{1}H-NMR spectrum of compound **1** (500 MHz, MeOH-d_4)

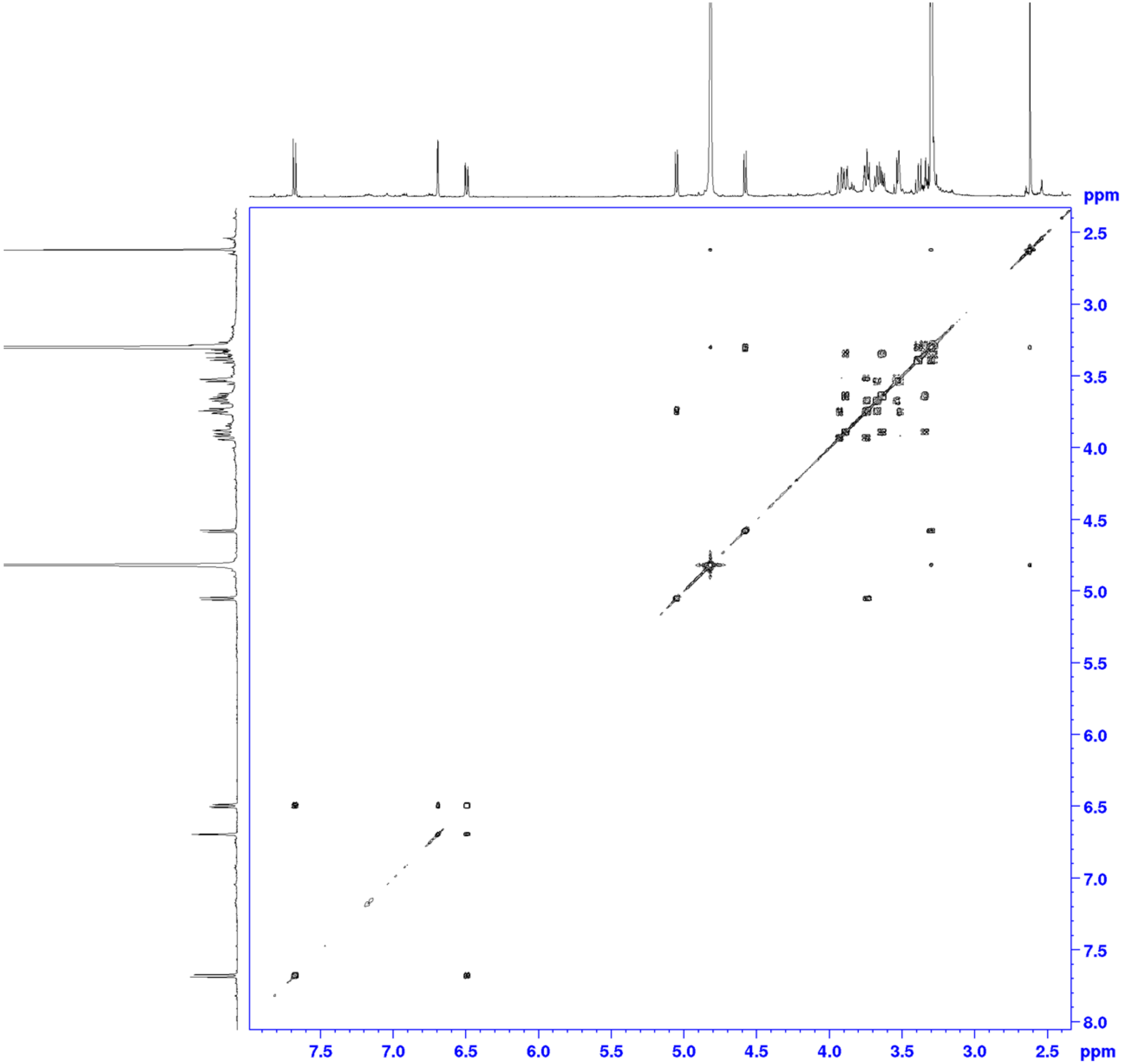

Figure S2. ^{1}H-^{1}H-COSY spectrum of compound **1** (500 MHz, MeOH-d_4)

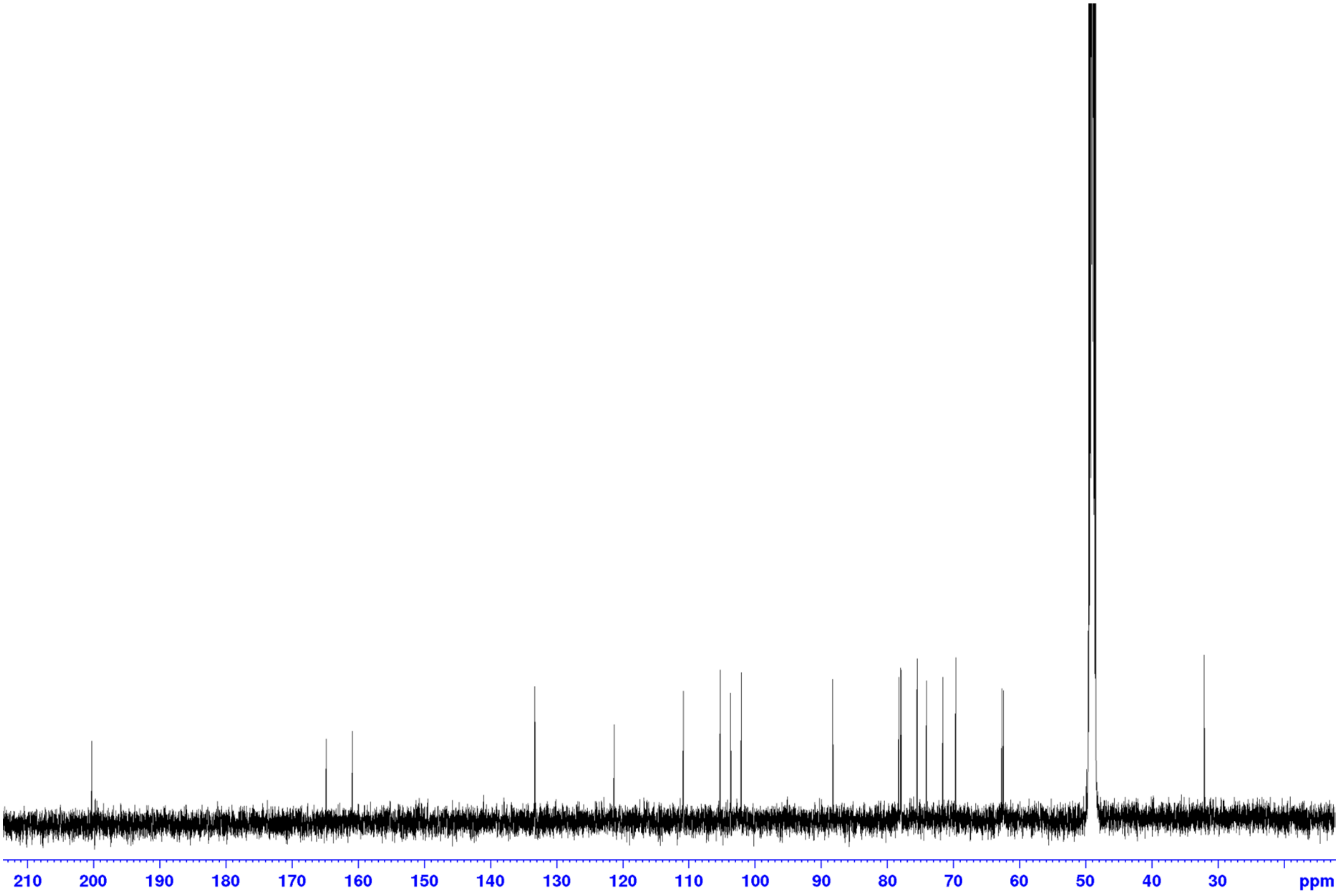

Figure S3. ^{13}C-NMR spectrum of compound **1** (126 MHz, MeOH-d_4)

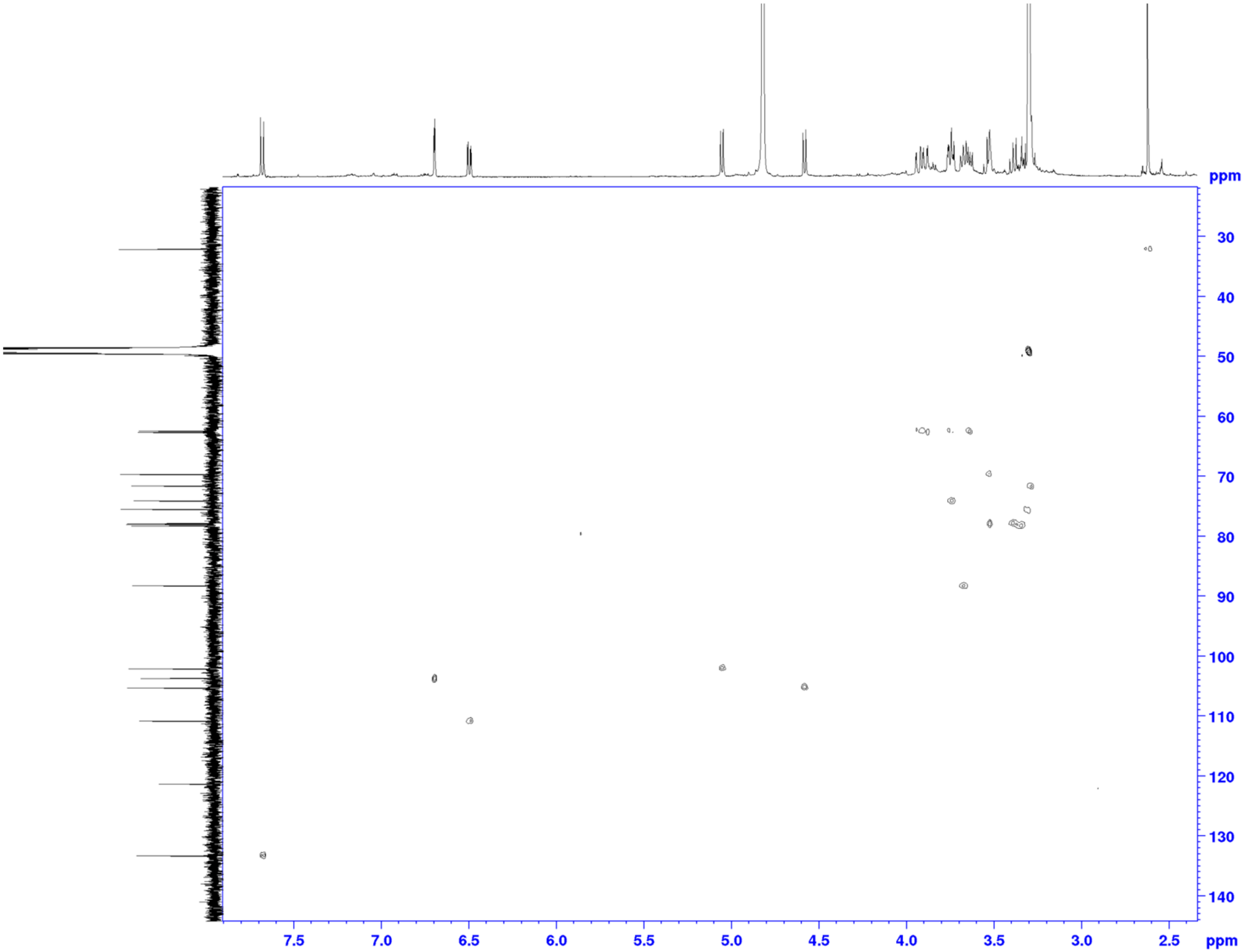

Figure S4. HSQC spectrum of compound **1** (500 MHz, MeOH-d_4)

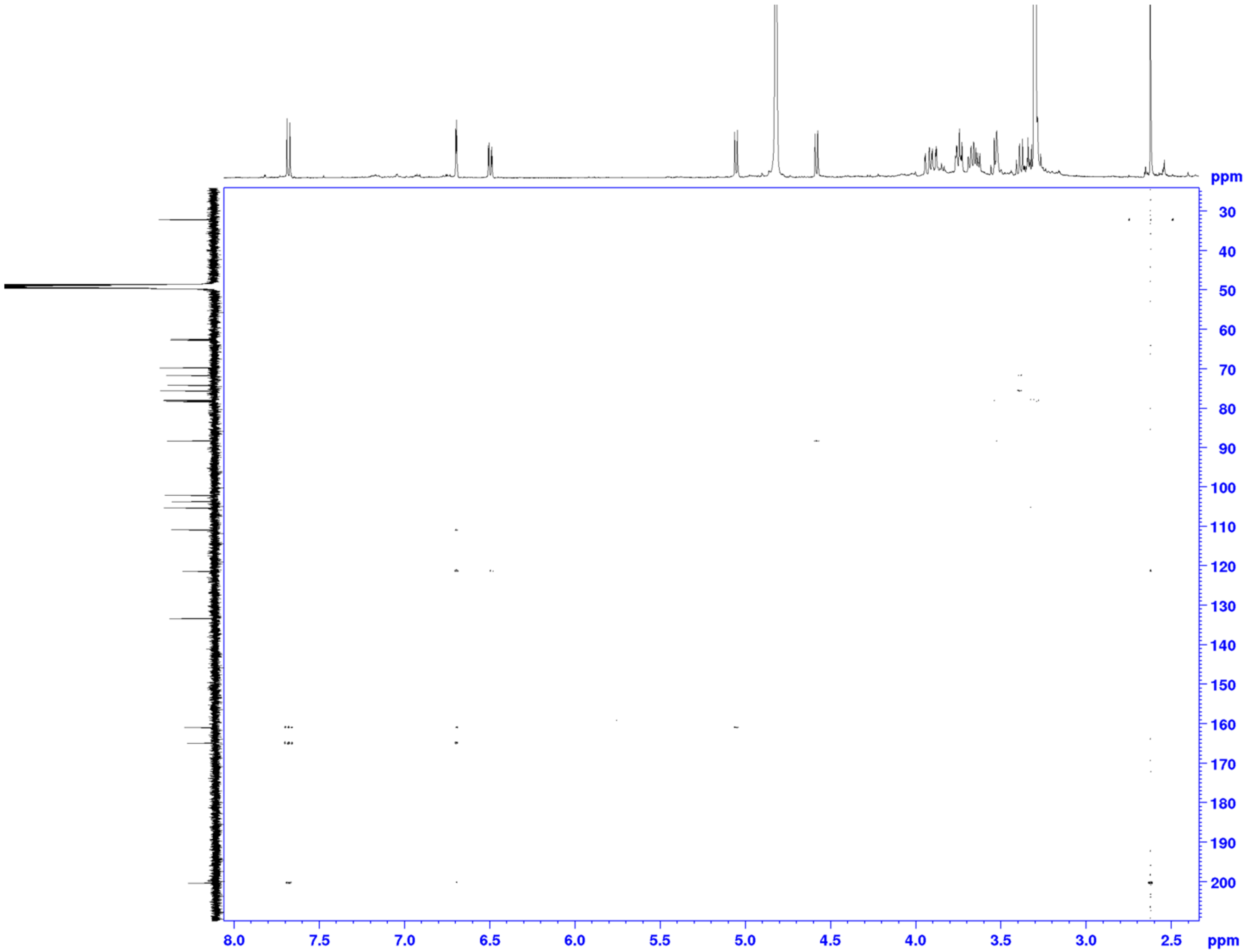

Figure S5. HMBC spectrum of compound **1** (500 MHz, MeOH-d_4)
